# Author Correction: Ballistic superconductivity in semiconductor nanowires

**DOI:** 10.1038/s41467-025-57863-x

**Published:** 2025-03-12

**Authors:** Hao Zhang, Önder Gül, Sonia Conesa-Boj, Michał P. Nowak, Michael Wimmer, Folkert K. de Vries, Jasper van Veen, Michiel W. A. de Moor, Jouri D. S. Bommer, David J. van Woerkom, Diana Car, Sébastien R. Plissard, Erik P. A. M. Bakkers, Marina Quintero-Pérez, Maja C. Cassidy, Sebastian Koelling, Srijit Goswami, Kenji Watanabe, Takashi Taniguchi, Leo P. Kouwenhoven

**Affiliations:** 1 QuTech, Delft University of Technology, 2600 GA Delft, The Netherlands; 2 Kavli Institute of Nanoscience, Delft University of Technology, 2600 GA Delft, The Netherlands; 3 Department of Applied Physics, Eindhoven University of Technology, 5600 MB Eindhoven, The Netherlands; 4 Faculty of Physics and Applied Computer Science, AGH University of Science and Technology, al. A. Mickiewicza 30, 30-059 Kraków, Poland; 5 Netherlands Organisation for Applied Scientific Research (TNO), 2600 AD Delft, The Netherlands; 6 Advanced Materials Laboratory, National Institute for Materials Science, 1-1 Namiki, Tsukuba, 305-0044 Japan; 7 Microsoft Station Q Delft, 2600 GA Delft, The Netherlands

Correction to: *Nature Communications* 10.1038/ncomms16025, published online 06 July 2017

The original version of this Article included the authors Kun Zuo and Vincent Mourik who wish to be removed from authorship. Consequently, the author affiliations for these authors have been removed from the ‘Authors and Affiliations’ section. The original version of the ‘Contributions’ statement, which read “*H.Z. and Ö.G. fabricated the devices, performed the measurements and analysed the data. S.C.-B. performed the TEM analysis. M.P.N. and M.W. performed the numerical simulations. K.Z., V.M., F.K.d.V., J.v.V., M.W.A.d.M., J.D.S.B., D.J.v.W., M.Q.-P., M.C.C. and S.G. contributed to the experiments. D.C., S.P. and E.P.A.M.B. grew the InSb nanowires. S.K. prepared the lamellae for the TEM analysis. K.W. and T.T. synthesized the h-BN crystals. L.P.K. supervised the project. All authors contributed to the writing of the manuscript*”, has been amended to read “*H.Z. and Ö.G. fabricated the devices, performed the measurements and analysed the data. S.C.-B. performed the TEM analysis. M.P.N. and M.W. performed the numerical simulations. F.K.d.V., J.v.V., M.W.A.d.M., J.D.S.B., D.J.v.W., M.Q.-P., M.C.C. and S.G. contributed to the experiments. D.C., S.P. and E.P.A.M.B. grew the InSb nanowires. S.K. prepared the lamellae for the TEM analysis. K.W. and T.T. synthesized the h-BN crystals. L.P.K. supervised the project. All authors contributed to the writing of the manuscript*”.

This has been corrected in both the PDF and HTML versions of the article.

