# Supplementary material for: Ballistic superconductivity in semiconductor nanowires
Source: Nat Commun. 2017 Jul 6;8:16025. doi: 10.1038/ncomms16025 (PMC5504288; doi:10.1038/ncomms16025)
Supplement: Supplementary Information [file ncomms16025-s1.pdf]

File name: Supplementary Information
Description: Supplementary Figures and Supplementary References

File name: Peer Review File
Description:

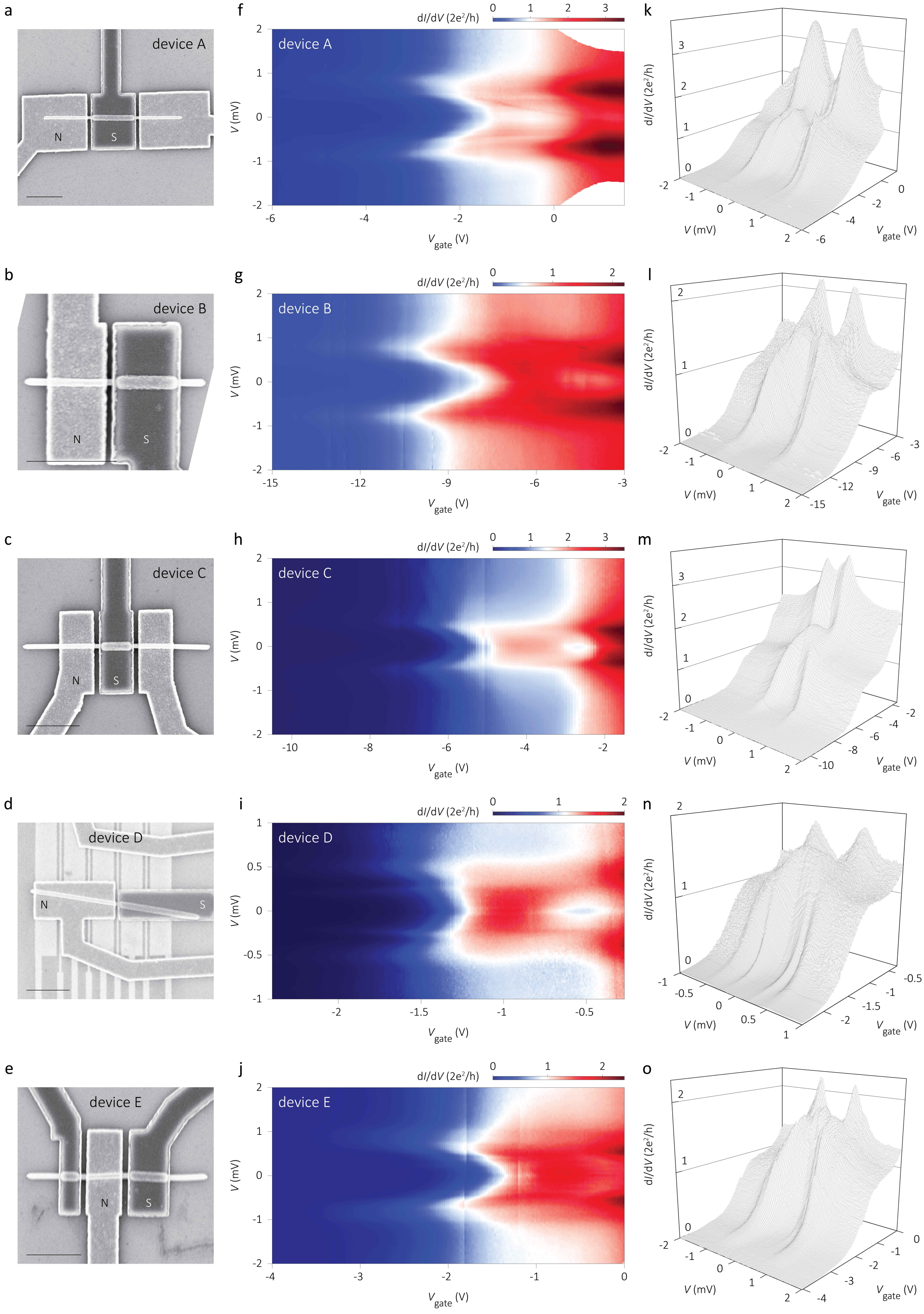

device A
device B
device C
device D
device E
N
S
dI/dV (2e²/h)
V (mV)
V gate (V)
a
b
c
d
e
f
g
h
i
j
k
l
m
n
o

Supplementary Figure 1 | **Electron micrographs and ballistic transport for all devices.** **a-e,** Electron micrographs of all presented devices. N indicates the normal metal lead, S the superconducting lead. For three terminal devices, unlabelled leads are kept floated throughout the measurements. Scale bars for all images indicate 1 μm. Data in Figure 3c (main text) is taken by sweeping the local gate underneath the uncovered wire section (see device D in panel d). **f-j,** Differential conductance of all devices in colour scale as a function of bias and gate voltage at zero magnetic field. **k-o,** Same as **f-j** but the differential conductance is on the vertical axis. All devices show quantized conductance, Andreev enhancement and an induced superconducting gap of 0.9 mV (A), 0.8 mV (B), 0.6 mV (C), and 0.52mV (D), 0.85 mV (E). Unintentional quantum dots are absent in all devices. Correspondence with the figures in main text: data in Figure 1 and Figures 4b-d taken on device A, Figures 2a-d on device B, Figure 2e and Figure 3d on device C, Figure 3c on Device D, Figure 4a on device E.

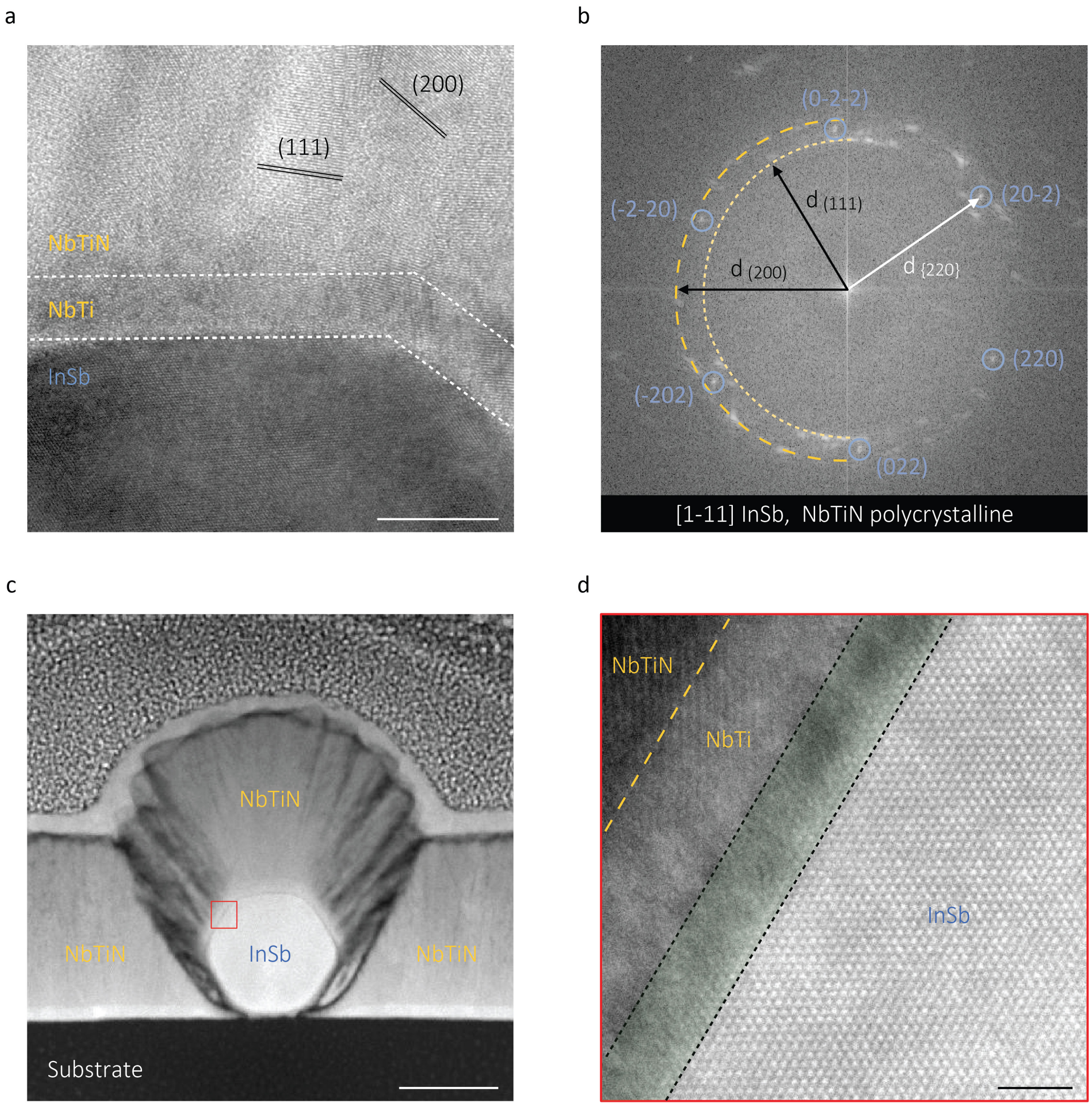

Supplementary Figure 2 | **Structure characterization and high-angle annular dark field analysis of the InSb-NbTi-NbTiN interface. a,** High-resolution TEM cross-sectional image obtained by aligning the InSb nanowire along the zone axis in the [111] orientation. Scale bar: 10 nm. **b,** Fast Fourier transform (FFT) corresponding to **a** revealing the presence of both InSb and NbTiN. The NbTiN contribution leads to the formation of several Bragg spots on concentric rings, which provides evidence of the existence of several grains with different orientations in the original image. Each ring with different radius corresponds to a particular value of the interplanar spacing, i.e., it is the result of the reflections associated to a specific family of planes. Two different d-spacings have been identified and measured: $d_{(111)} = 0.249\,\mathrm{nm}$ and $d_{(200)} = 0.213\,\mathrm{nm}$. They are indicated with yellow dashed lines in **b**. From these values, it is possible to determine the NbTiN

lattice parameter which we obtain to be 0.430 nm. On the other hand, the six {220} reflections, indicated by a blue circle in the FFT, arise from the InSb region. From the calculated interplanar value of $d_{\{220\}} = 0.230\,\mathrm{nm}$ we can estimate the InSb lattice parameter to be around 0.649 nm. The obtained lattice parameters for both NbTiN and InSb are in good agreement with the respective values reported in the literature. **c,** High-angle annular dark field (HAADF) scanning transmission electron microscopy (STEM) image of the device cross-section. Scale bar: 50 nm. **d,** Atomic resolution HAADF-STEM image of the interface for the region marked by the red square in **c**. Scale bar: 2 nm. The highlighted area in green indicates the 2 nm deep NbTi segregation into the InSb core. This effect is in good agreement with the EDX line scan, see Figure 1f (main text), which shows the NbTi region to be segregated 2 nm deep into the InSb core on the {220} facets.

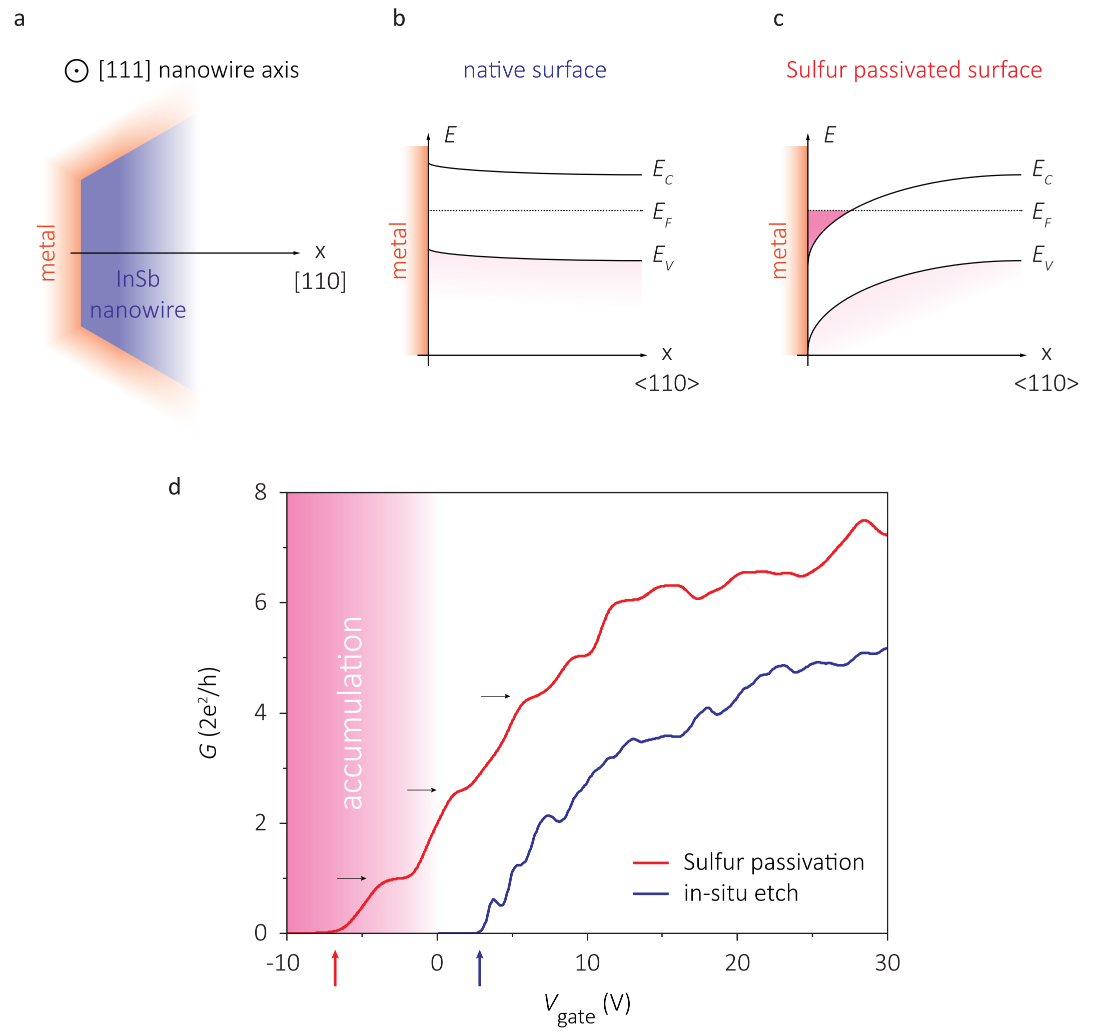

Supplementary Figure 3 | **Inclusion of Sulfur creates surface accumulation by band bending. a,** A schematic of the device cross-section showing both the nanowire and the contact metal. Nanowire axis is along [111] and the hexagonal facets have <110> orientation. **b,** Band diagram for an InSb surface with <110> orientation. Fermi level at the surface lies closer to the valence band[1]. **c,** Band diagram for an InSb surface with <110> orientation passivated with Sulfur-based solution. Inclusion of Sulfur at the surface counteracts the depletion and pins the Fermi level in the conduction band. This creates an electron-rich nanowire surface. **d,** Gate voltage response of nanowire devices with different contact preparation. A finite conductance for negative gate voltages indicates electron accumulation, highlighted in pink. Blue curve is taken from a device with contacts prepared using in-situ Ar plasma etch, red curve from a device with contacts prepared using Sulfur passivation. Both devices have identical geometries with a channel length of 150 nm. Devices with Sulfur passivated contact areas exhibit a negative threshold voltage (red arrow), in contrast to the devices with in-situ plasma etched contact areas (blue arrow). This typical characteristic measured on hundreds of nanowire devices indicates the doping effect of Sulfur inclusion at the InSb nanowire surface. Black arrows indicate the 1st, the 3rd, and the 5th quantized con-

ductance plateau[2]. Upper plateaus appear at lower conductance values due to a finite contact resistance which is not subtracted from the conductance traces shown here. We also find that Sulfur passivated contacts show lower contact resistances than in-situ etched contacts, which can be seen from the difference in saturation conductance of both traces at high gate voltages.

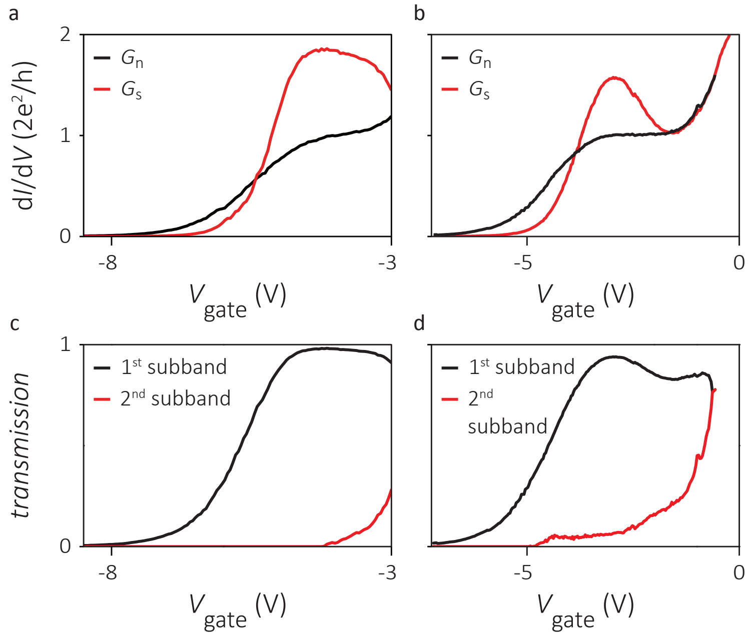

Supplementary Figure 4 | **Subband mixing. a, b,** The panels show the measured data taken from the main text, **a** from Figure 2e and **b** from Figure 3c. **c, d,** The panels show the transmission T of the first two subbands extracted from **a** and **b**, respectively, using $G_{\rm n} = 2e^2/h \sum T_i$ and $G_{\rm s} = 4e^2/h \sum T_i^2/(2 - T_i)^2$. When the transmission of the second subband becomes finite, the conductance modes mix, decreasing the Andreev enhancement. This effect shows up as a dip in $G_{\rm s}$ when plotted as a function of gate voltage $V_{\rm gate}$.

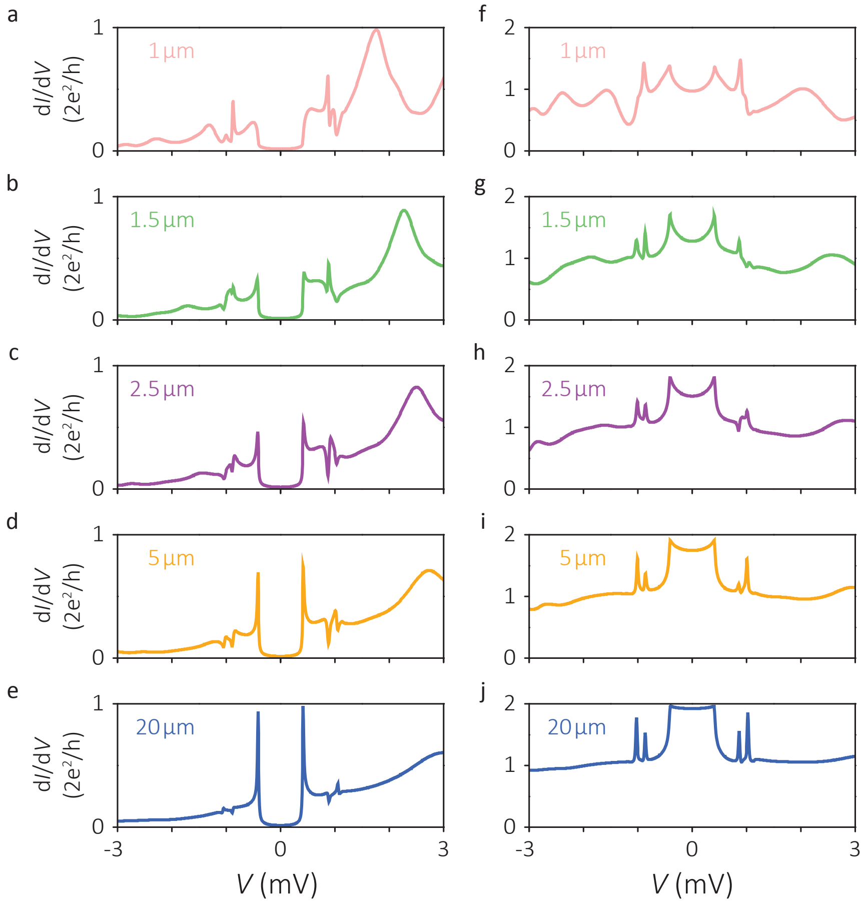

Supplementary Figure 5 | **Simulated conductance for varying mean free paths. a-e,** The tunnelling conductance for different disorder strengths with mean free path l_e ranging from $1\,\mu$m to $20\,\mu$m. **f-j,** The conductance in the plateau regime for different disorder strengths with mean free path l_e ranging from $1\,\mu$m to $20\,\mu$m. For short l_e, the Andreev enhancement is lower than that observed in our measurements and disorder-induced conductance fluctuations dominate the conductance for above-gap bias which we do not observe in our measurements. A good correspondence with the measurements is found for $l_e > 2.5\,\mu$m. We note that the details of the peak-dip structure in the Andreev conductance depend on several additional parameters of the system (e.g. barrier smoothness or disorder realization). In contrast, we find the conductance fluctuations above the gap to depend mainly on disorder strength, so that the combined analysis can be used as an estimate for the mean free path. The four symmetric peaks outside the gap around $V \sim \pm 1\,$mV indicate the induced superconducting gap for the higher two subbands (three subbands are occupied in total). Asymmetric conductance for different bias polarity is due to energy dependent transmission through the tunnel barrier, i.e., for positive bias V, effective QPC potential is lower.

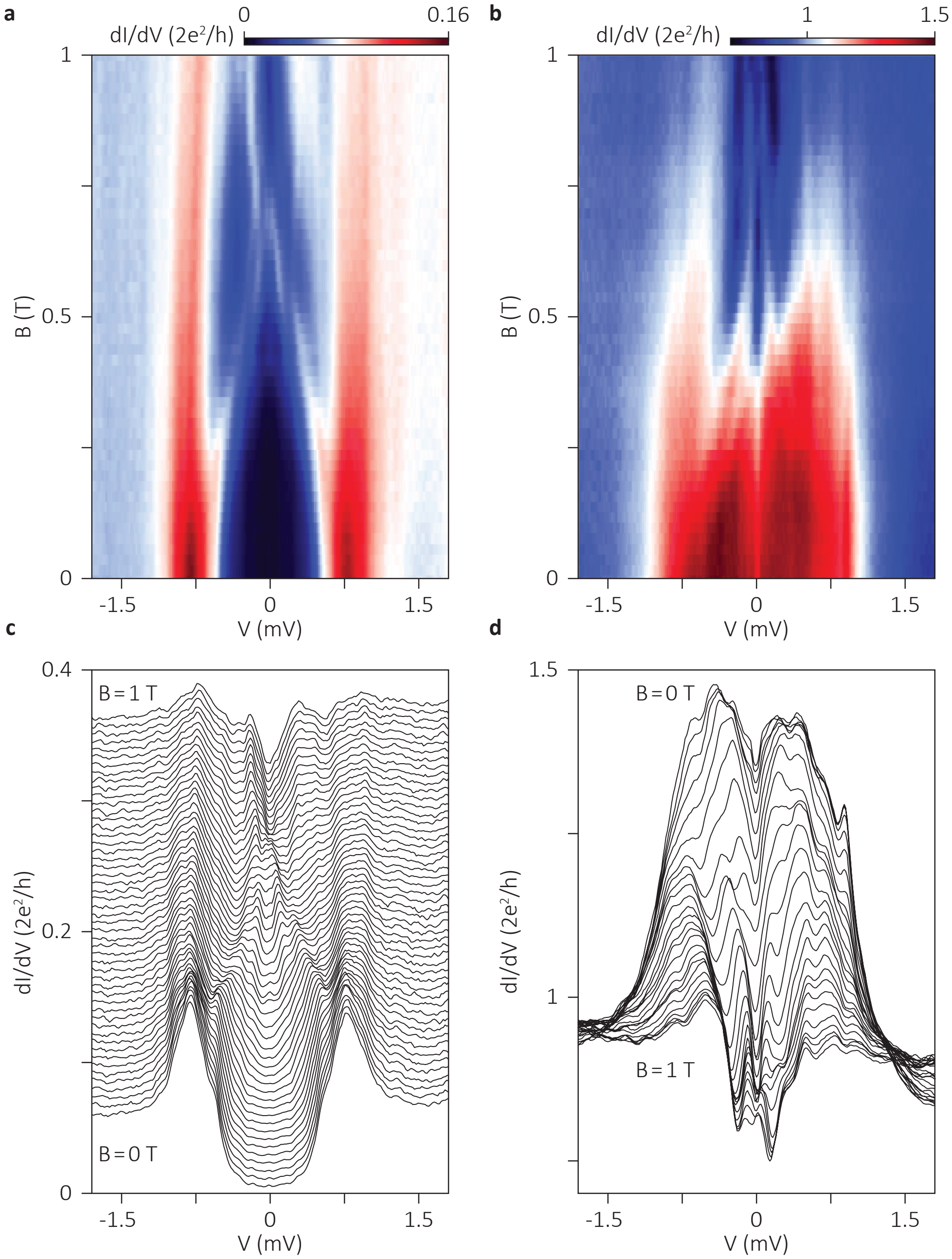

Supplementary Figure 6 | **Magnetic field dependence of the induced gap and Andreev enhancement. a,** Differential conductance $\mathrm{d}I/\mathrm{d}V$ of device B as a function of magnetic field B along the nanowire when the device is in the tunneling regime (gate voltage = $-12\,\mathrm{V}$). We find an induced superconducting gap of $0.8\,\mathrm{mV}$ at zero magnetic field. Increasing magnetic fields increase the subgap conductance but a gap feature can be identified at least up to 1 Tesla, indicating that the NbTiN leads are still superconducting. **b,** Differential conductance $\mathrm{d}I/\mathrm{d}V$ of the same device as a function of magnetic field B (along the wire axis) when the device is in the plateau regime (gate voltage = $-6\,\mathrm{V}$). At zero magnetic field, we find an enhancement of conductance up to a factor of ~ 1.5 for subgap energies due to Andreev reflection. Increasing magnetic fields have a

negligible effect on the above-gap conductance. In contrast, the Andreev enhancement is suppressed at 1 Tesla, a magnetic field at which the NbTiN leads are still superconducting. **c,** Line cuts from the data in **a** with vertical offsets $0.006 \times 2e^2/h$. **d,** Line cuts from the data in **b** without offset.

SUPPLEMENTARY REFERENCES

[1] Gobeli, G. W. & Allen, F. G. Photoelectric properties of cleaved GaAs, GaSb, InAs, and InSb surfaces; comparison with Si and Ge. *Phys. Rev.* **137**, A245 (1965)

[2] Kammhuber, J. *et al.* Conductance quantization at zero magnetic field in InSb nanowires. *Nano Lett.* **16**, 3482 (2016)
